# The phenotypic spectrum of *PTCD3* deficiency

**DOI:** 10.1002/jmd2.12424

**Published:** 2024-05-27

**Authors:** Baiba Lace, Eissa Faqeih, Namik Kaya, Zita Krumina, Johannes A. Mayr, Ieva Micule, Nathan Thompson Wright, Melanie T. Achleitner, Hanan AlQudairy, Sander Pajusalu, Janis Stavusis, Pawel Zayakin, Inna Inashkina

**Affiliations:** ^1^ Riga East Clinical University Hospital Riga Latvia; ^2^ Institute of Clinical and Preventive Medicine, University of Latvia Riga Latvia; ^3^ Section of Medical Genetics Children's Specialist Hospital, King Fahad Medical City Riyadh Saudi Arabia; ^4^ Translational Genomics Department MBC: 26, Centre for Genomic Medicine Riyadh Saudi Arabia; ^5^ Department of Biology and Microbiology Riga Stradiņš University Riga Latvia; ^6^ University Children’s Hospital, Laboratory Salzburger Landeskliniken Universitaetsklinikum of the Paracelsus Medical University Salzburg Salzburg Austria; ^7^ Department of Medical Genetics and Prenatal Diagnostics Children's University Hospital Riga Latvia; ^8^ Department of Chemistry and Biochemistry James Madison University Harrisonburg Virginia USA; ^9^ Department of Clinical Genetics, Genetics and Personalized Medicine Clinic Tartu University Hospital Tartu Estonia; ^10^ Department of Genetics and Personalized Medicine Institute of Clinical Medicine, Faculty of Medicine, University of Tartu Tartu Estonia; ^11^ Latvian Biomedical Research and Study Centre Riga Latvia

**Keywords:** Leigh‐like syndrome, pentatricopeptide repeat, PTCD3

## Abstract

The *PTCD3* gene product (protein PTCD3 or MRPS39) forms the entry channel of the mitochondrial small ribosomal subunit and binds to single‐stranded mRNA. Here, we expand on the clinical manifestations of *PTCD3* pathogenic variants by describing an early‐onset patient with Leigh‐like syndrome and two patients with milder form of disease, with combined oxidative phosphorylation deficiency. A 34‐year‐old male and his 33‐year‐old sister both have horizontal nystagmus, pronounced rough tremor, truncal ataxia, dysmetria, spasticity and hyperreflexia. The basal respiration rate decreased significantly for the male patient and his mother (*p* < 0.0001) compared to the controls. The whole genome sequencing analysis revealed two heterozygous variants in the *PTCD3*: c.1182T>A, p.(Tyr394Ter) and c.805C>T, p.(His269Tyr). Tyr394Ter variant ablates the C‐terminal half of the protein, including a significant portion of the central fold. In silico modelling for the variant His269Tyr shows that the inclusion of the slightly larger tyrosine sidechain is well tolerated, with no significant change in either the position or the movement of the surrounding area. The third case is a 9‐year‐old boy, who has a global developmental delay, central hypotonia, hyperreflexia and abnormal MRI. *PTCD3* pathogenic variant c.538+4A>G was identified by whole exome sequencing. To test the variant's effect on splicing, an RT‐PCR experiment was performed, which revealed skipping of an out‐of‐frame exon 7.

**Synopsis**

Loss of function genetic variants in the *PTCD3* gene are usually associated with severe progressive disease with neurodevelopmental problems and early death; here we describe also a new and a milder form of this disease—combined oxidative phosphorylation deficiency, which presents with optic atrophy, ataxia and neuropathy.

## 1 INTRODUCTION

Mitochondrial ribosomes or mitoribosomes, similar to cytoplasmic ribosomes, have a large 39S subunit (mt‐LSU) and a small 28S subunit (mt‐SSU).^1^ The product of the pentatricopeptide repeat domain 3 (*PTCD3*) gene contributes to the function of the mitochondrial small ribosomal subunit. PTCD3 (or mS39) forms the entry channel of the mitoribosome and binds to single‐stranded mRNA.^2^ The entire C‐terminal two‐thirds of PTCD3 consists of multiple pentatricopeptide repeats (PPR), which are necessary for interaction with RNA. PPR‐containing proteins are rare in mammalian cells; however, there is growing evidence of their, particularly PTCD3, involvement in mitochondrial metabolism.^3^ PTCD3 is expressed in all tissues.^4^

The *PTCD3* knock‐out mutant of Drosophila is prenatally lethal, with defects in mitochondrial function and neuronal development.^5^ Similarly, *PTCD3* loss‐of‐function mice exhibit neurodevelopmental defects and early lethality.^6^ Currently, there are three familial cases reported with Leigh or Leigh‐like syndrome (progressive early necrotising encephalopathy) due to loss‐of‐function variants of the *PTCD3*, which cause reduced expression of the PTCD3 protein and altered mitochondrial respiration.^7^, ^8^ The first described patient presented with early respiratory failure, progressive neurodegenerative disease and Leigh‐like brain magnetic resonance lesions.^7^ Three additional patients from two families had developmental delay, seizures, respiratory insufficiency, optic nerve hypoplasia, abnormal brain magnetic resonance imaging and died in the first or second decade.^8^

Here, we expand on the clinical descriptions of *PTCD3* variants by presenting one early‐onset Leigh‐like syndrome patient and two combined oxidative phosphorylation deficiency patients, each cohort with unique *PTCD3* pathogenic variants.

## 2 MATERIALS AND METHODS (SUPPLEMENT 1 METHODS IN DATA S1)

### 2.1 Patients

#### 2.1.1 Cases 1 and 2

A 34‐year‐old male (case 1) and his 33‐year‐old sister (case 2) both have horizontal nystagmus, pronounced rough tremor, truncal ataxia, dysmetria, spasticity and hyperreflexia. Both are legally blind, retaining only light perception, experiencing mild cognitive decline and being non‐ambulatory. They are from a non‐consanguineous family without a remarkable family history. Both patients were born from uncomplicated pregnancies and their early development was normal. Vision problems began at the age of 2 years, when the clinical diagnosis of horizontal nystagmus was established for case 1 with progression over the next 2 years leading to optical nerve atrophy. An instability and a progressive gait abnormality at the age of 6 years led to a neurology consult, where dysmetria, severe tremor and truncal ataxia were described. EEG, echocardiography and hearing tests did not reveal any pathology, but EMG/NCV showed motor polyneuropathy. Metabolic investigations of amino acids in blood and urine, and oligosaccharides in urine, as well as transferrin isofocusing were normal, except for a mild elevation of lactic acid 2.9–3.2 mmol/L (reference: 2.4 mmol/L). Urinary organic acid analysis were within reference range for both of them, except 3‐OH propionic acid in case 1 (169.98 mM/M creat; N 0–20) and in case 2 it was increased to 58.63 mM/M creat (N 0–20).

A slightly milder presentation of identical clinical symptoms affected his sister's health (case 2), and genetic testing was initiated on the hypothesis of mitochondrial pathology or hereditary ataxia for both. The results of mtDNA sequencing, Friedreich ataxia repeat expansion analysis, NGS ataxia gene panel (case 1), nucleotide repeat expansion analysis for all known types of spinocerebellar ataxia (case 1) and whole exome sequencing (case 2) did not reveal the cause of the disease. Both patients received education at a special school for the visually impaired.

Brain magnetic resonance imaging for case 1 was normal at age 20, however, due to the severe tremor, it was not possible to repeat it recently without sedation, which was refused by the patient and his family (Videos S1 and S2).

#### 2.1.2 Case 3

The third case was identified using the GeneMatcher tool.^9^ The patient is a 9‐year‐old boy from a full‐term uneventful pregnancy, born through spontaneous vaginal delivery, with a birth weight of 3 kg. His parents are double first cousins with no familial history of similar conditions. The parents have one healthy daughter, and the mother has experienced previously three spontaneous abortions. The patient has had a global developmental delay since birth with no seizures. At 9 years of age, he was able to sit and crawl, babble and make sounds. He has dysphagia with frequent episodes of choking and no sphincter control. The clinical evaluation revealed that weight and height were below the third percentile with normal head circumference. He has a myopathic face, bilateral squints and nystagmus, bushy eyebrows and a sialorrhea. He showed central hypotonia with peripheral hypertonia and hyperreflexia. No organomegaly was observed. Laboratory investigation revealed normal results of blood lactate, ammonia, creatine phosphokinase, cholesterol and electrolytes in variable ages (2, 6 and 7 years). Organic acids in urine by tandem MS were done several times and only showed border‐low free carnitine. Brain magnetic resonance imaging (MRI) at 6 years of age showed a bilateral symmetrical globus pallidus signal intensity with an elevated lactate peak. Repeated brain MRI at 8 years of age showed cystic changes, gliosis and volume loss involving bilateral posterior putamina with high signal intensity in T2 and FLAIR, low signal intensity in T1 and no associated susceptibility effect or diffusion restriction. The performed MR spectroscopy shows no other abnormal metabolites detected, except lactate peak. The brainstem, vermis and cerebellum appear unremarkable. These clinical manifestations align with a Leigh‐like syndrome.

## 3 RESULTS AND DISCUSSION

**FIGURE 1:**
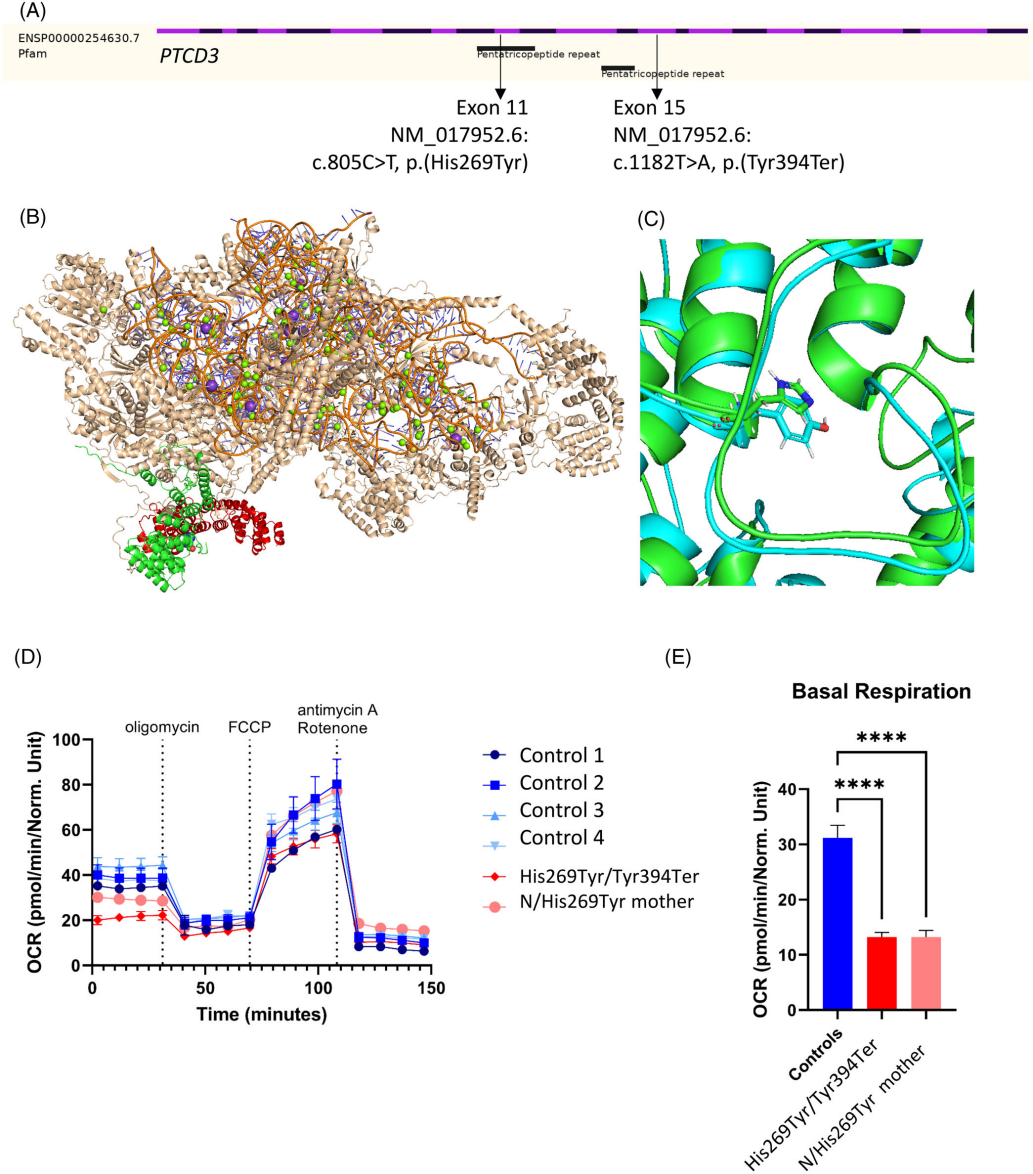
*PTCD3* variants c.805C>T, p.(His269Tyr) and c.1182T>A, p.(Tyr394Ter) analysis. (A) *PTCD3* gene structure with variant localisation in exon 11th and 15th. (B) Cryo‐EM structure of the mt‐SSU (PDB: 6GAZ) showing PTCD3 (green/red). Red denotes the part of the structure c‐terminal to the Tyr394Ter mutation. (C) MD of WT (green) and His269Tyr (blue) PTCD3 show similar backbone and sidechain placement. (D) Oxygen consumption rate (OCR) were measured with XF96 extracellular flux analyser (Seahorse, Agilent) in fibroblasts of two *PTCD3* individuals and controls. OCR in pmol/min/Norm. Unit. The data show mean values ± SEM (number of replicates: patient = 8; control = 17). (E) The data show the mean values ± SEM of basal respiration (number of replicates: patient = 8; control = 17), ****p < 0.0001.

Two variants in the *PTCD3* were identified via whole genome sequencing (Supplement 1 Methods in Data S1) in cases 1 and 2. The results were validated by Sanger sequencing, followed by family segregation analysis, which confirmed their *trans* position in the gene. The first variant NM_017952.6: c.1182T>A, p.(Tyr394Ter) hereinafter Tyr394Ter is localised in exon 15 (Figure 1A). This nonsense change has a high pathogenicity metascore and has not been previously reported in GnomAD. The CADD score of this variant is 36.^10^ Semi‐automated ACMG classification attributes PVS1 and PM2 criteria, identifying it as likely pathogenic.^11^ This pathogenic variant ablates the C‐terminal half of the protein (red; Figure 1B), including a significant portion of the central fold; therefore, proteins harbouring this variant cannot adopt a native fold and cannot be correctly incorporated into the mitochondrial ribosome. Western blot analysis of PTCD3 does not differ between patients and controls, concluding that the Tyr394Ter variant does not cause nonsense‐mediated decay (Supplement 1 in Data S1, Figure 1).

The second is a missense variant NM_017952.6: c.805C>T, p.(His269Tyr) hereinafter His269Tyr, which is the first nucleotide in exon 11 and within the PPR domain. The allele frequency for this variant in the GnomAD database is 0.000004.^12^ The histidine in this position is conserved in fish and mammals. The CADD score is 21.50.^10^ A series of previously reported cryo‐EM structures^1^, ^13^, ^14^, ^15^, ^16^ show that PTCD3 is located on the periphery of the large mitochondrial ribosome subunit and His269 is positioned towards the hydrophobic centre of the protein (Figure 1C). In silico modelling shows that the inclusion of the slightly larger tyrosine sidechain is well tolerated, with no significant change in either the position or the movement of the surrounding area. Therefore, it is unlikely that this pathogenic variant influences either the PTCD3 structure of or target binding. The rarity of the variant allowed us to use the PM2 ACMG classification and classify it as the variant of unknown significance.^11^

The oxygen consumption rate (OCR) was measured with the XF96 extracellular flux analyser (Seahorse, Agilent) in fibroblasts of case 1 individual and his unaffected mother in comparison with controls (Figure 1D). The basal respiration rate decreased significantly for the case 1 patient and his mother (*p* < 0.0001) compared to controls (Figure 1D,E). OCR before the addition of oligomycin showed a reduction (~20 pmol/min/Norm. Unit) for the case 1 sample, compared to the mother and controls (~30–50 pmol/min/Norm. Unit). These results suggest that both *PTCD3* variants Tyr394Ter and His269Tyr affect protein function, despite our modelling predictions. One of the identified variants Tyr394Ter meets the pathogenic criteria. In a patient this terminating variant is responsible for the significantly decreased basal respiration rate compared to controls. The similar results were observed in his unaffected mother, who is a carrier of the His269Tyr variant, concluding that both heterozygous unaffected and affected carriers exhibit a lower OCR. However, this lower rate is not enough to cause the disease, as there is no significant difference between the affected patient and his unaffected mother.

Furthermore, in silico, protein analysis confirms that the Tyr394Ter terminating variant prevents the binding of the PTCD3 protein to mt‐SSU. The His269Tyr variant and the non‐Leigh‐like clinical presentation add to the complexity of cases 1 and 2. The genetic change His269Tyr has been classified as a variant of uncertain significance. Additional information obtained by us – being in *trans* position with a pathogenic variant and fitting with family segregation studies allows us to reclassify it as likely pathogenic.

**FIGURE 2:**
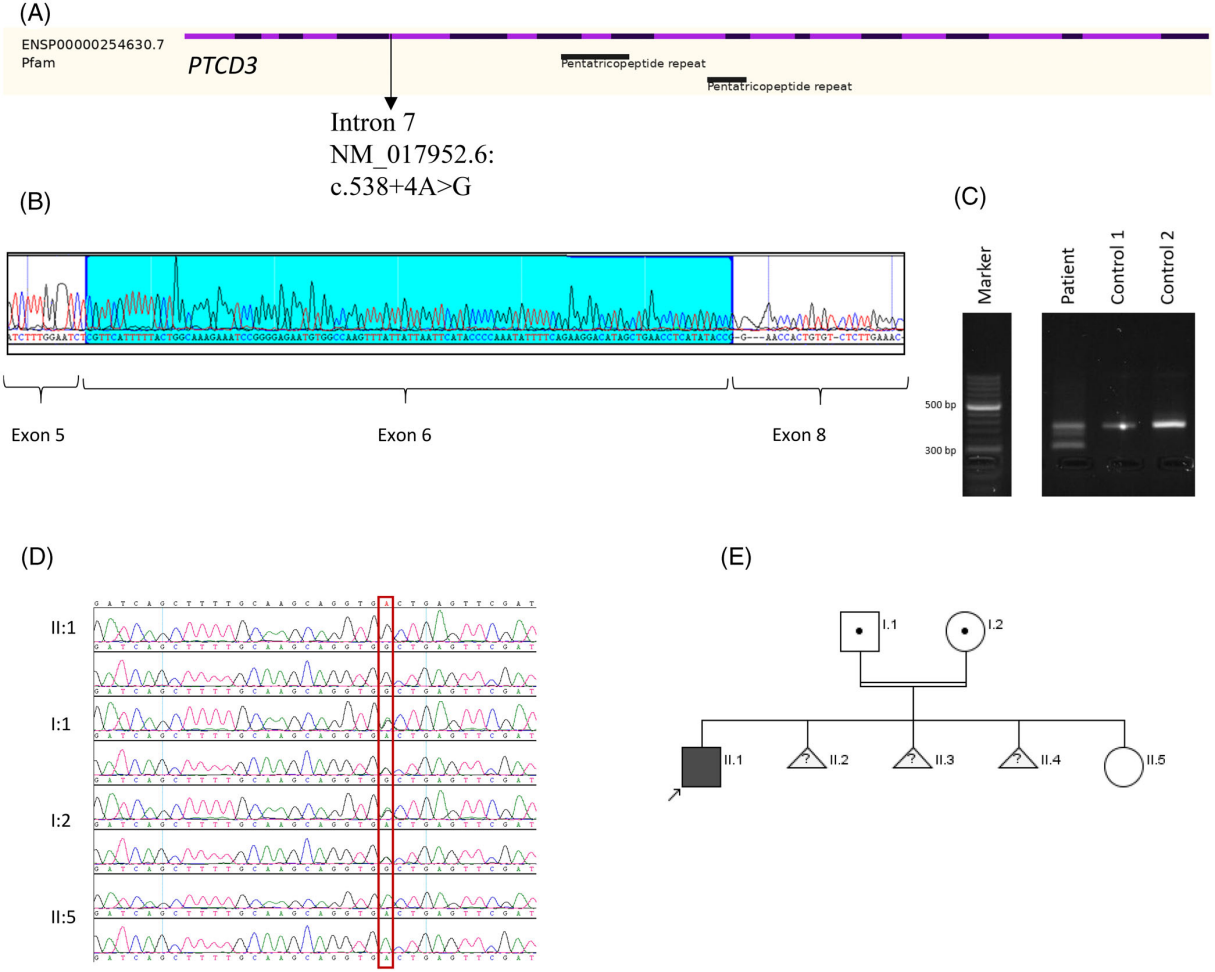
*PTCD3* variant NM_017952.5:c.538+4A>G analysis. (A) *PTCD3* gene structure with variant localisation in intron 7. (B) Band from agarose gel was sequenced using Sanger sequencing, which revealed skipping of Exon 7 (124 bp). (C) The band of the aberrant transcript was cut out using a sterile blade from 2% agarose gel. DNA extraction was performed using QIAquick Gel Extraction Kit (QIAGEN). (D) Familial segregation studies confirming homozygous c.538+4A>G variant in proband, heterozygous parents and homozygous normal unaffected sister. (E) Family pedigree.

In case 3, WES revealed a homozygous variant of uncertain significance in intron 7 of the *PTCD3* NM_017952.6: c.538+4A>G, which was confirmed by Sanger sequencing. This variant was also absent from the gnomAD database.^12^ The CADD score is 21.30.^10^ Splicing Prediction Pipeline algorithm predicted a high risk of alteration for the splice site (98.41%).^17^ Segregation analysis showed that the parents are heterozygous carriers, and the non‐affected normal sister is homozygous for the normal variant. To test the variant's effect on the splicing, an RT‐PCR experiment was performed, which revealed the skipping of exon 7 (124 bp) (Figure 2).

Genetic changes that affect splice sites are frequently not disease‐associated due to residual correct splicing events; many pathogenic variants suspected of affecting splice sites are classified as of uncertain significance or benign variants. However, *PTCD3* seems to be particularly vulnerable to pathological splice site errors, in spite of pLI‐score of 2.487e−22 (ranked 18 512 most intolerant of LoF pathogenic variants out of 19 704 genes under study).^18^ A Leigh‐like syndrome has previously been shown to be caused by altered *PTCD3* splicing; two missense variants (c.1918C>G and c.902C>T) alter splicing in exons 12 and 23, respectively, in these patients.^8^

In addition to confirming the alterations in the splicing machinery as one of the most important pathogenetic mechanisms of the *PTCD3*, we add the report of the homozygous intronic variant c.538+4A>G as a function‐altering pathogenic variant. This genetic change abolishes the consensus splice site and causes exon 7 skipping, which is essential for all protein‐coding transcripts in all tissues.^4^ The clinical symptoms of case 3 meet the criteria of Leigh‐like syndrome: a progressive neurodegenerative disease with abnormal brain magnetic resonance imaging that affects the basal ganglia with an elevated lactic acid signal.

**FIGURE 3:**
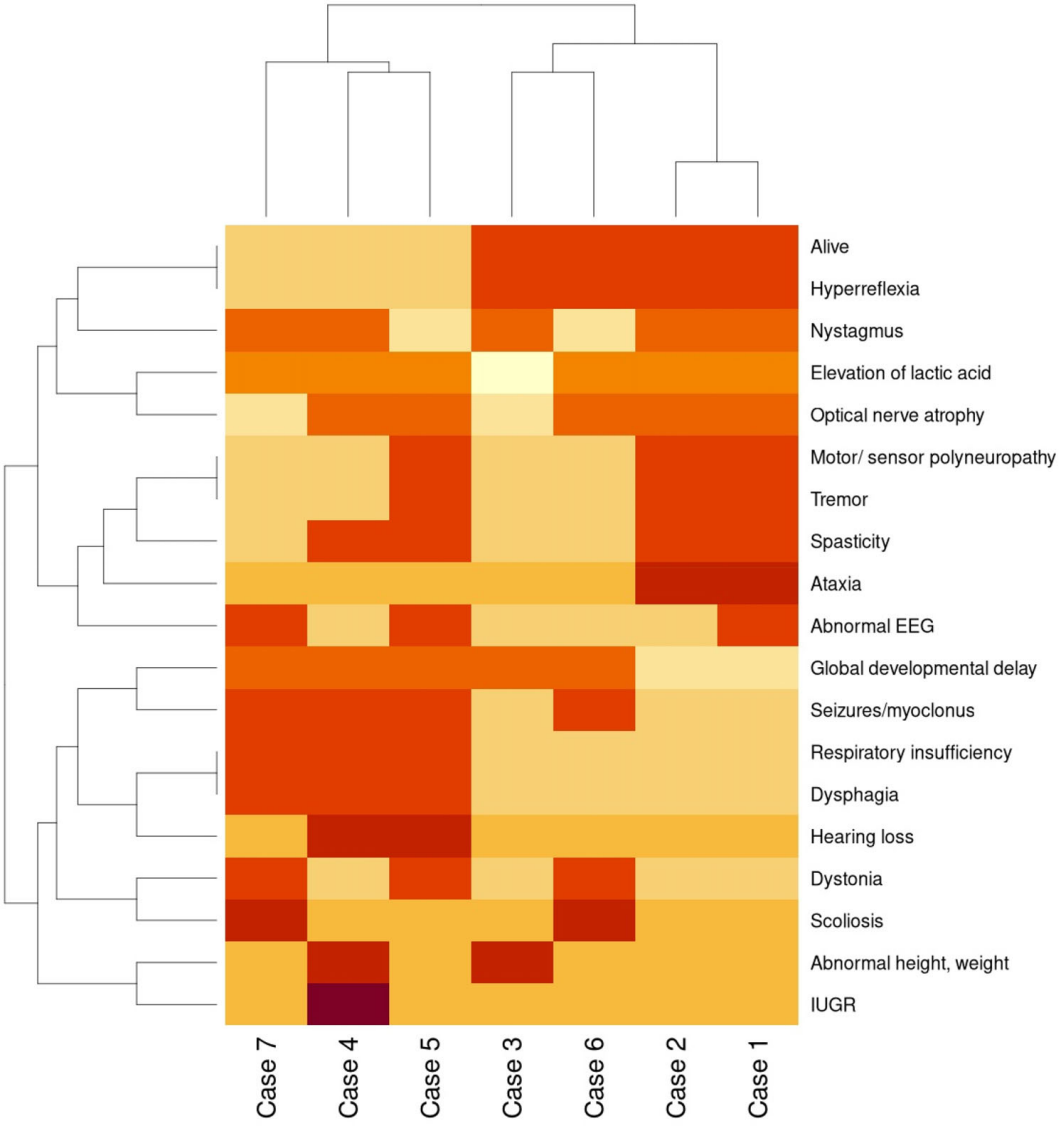
Heat map of clinical symptoms in PTCD3 patients. Cases 1, 2 and 3 described here in the current report. Case 4, patient with *PTCD3* pathogenic variants c.415‐2A>G and c.1747_1748insCT (p.Phe583Serfs*3).^7^ Case 5, patient 1 with *PTCD3* pathogenic variants c.1453‐1G>C and c.1918C>G.^8^ Case 6, patient 2.1 with *PTCD3* pathogenic variants c.710del and c.902C>T.^8^ Case 7, patient 2.2 with *PTCD3* pathogenic variants c.710del and c.902C>T.^8^

The phenotype of two patients (cases 1 and 2) presented here has been suggestive of mitochondrial pathology since their early age. The symptoms of both siblings come from multiple systems that resemble a combined oxidative phosphorylation deficiency. The heat map was generated with clinical features of previously and currently published patients, including cases 1 and 2 (Figure 3), and showed optic atrophy and nystagmus in six of seven patients. Defects in mitochondrial replication, RNA transcription and translation initiate early‐onset optic atrophy, as these cells are highly vulnerable to mitochondrial dysfunction.^19^ Global developmental delay was described in all Leigh‐like patients, except our presented cases 1 and 2, who have only mild cognitive decline. Severely affected *PTCD3* patients have spasticity, abnormal EEG, seizures, myoclonus, dystonia, dysphagia, polyneuropathy (Figure 3, cases 4, 5 and 7) and respiratory deficiency. This is a severe progressive disease, with neurodevelopmental problems and early death caused by loss of function genetic variants in *PTCD3*.^7^, ^8^ Cases 1 and 2 patients have ataxia, tremor, paraplegia, spasticity and polyneuropathy. We hypothesise that *PTCD3* missense pathogenic variants lead to a milder form of combined oxidative phosphorylation deficiency, probably due the higher level of residual PTCD3.

*LRPPRC* is another gene from PPR repeat family and bears similarity to the *PTCD3*. Pathogenic variants in *LRPPRC* are responsible for causing the French‐Canadian variant of Leigh syndrome. Due to the founder pathogenic variant NM_133259.4: c.1061C>T p.(Ala354Val), the French‐Canadian Leigh syndrome is common in the Saguenay‐Lac‐Saint‐Jean region of Quebec province in Canada. It represents a distinct phenotype characterised by severe acute metabolic and/or neurological crises resulting in a mortality rate that exceeds 80%. However, survivors beyond the age of 13 years have not encountered further metabolic or neurological crises. Additionally, these individuals exhibit symptoms such as truncal ataxia, mild intention tremors and developmental as well as language delays.^20^ This group of patients with variants in *LRPPRC* presents a similar phenotype as our described cases 1 and 2.

PTCD3 is only peripheral to the small ribosomal subunit. However, PTCD3 binds to ribosomal initiation factors,^21^ regulates mitochondrial translation^22^ and its ablation causes metabolic defects. This is likely the molecular aetiology of PTCD3‐linked neurologic and metabolic diseases, including those presented here.

## 4 CONCLUSIONS

Novel *PTCD3* loss‐of‐function intronic variant c.538+4A>G was described. This genetic change abolishes the consensus splice site and causes exon 7 skipping, which is essential for all protein‐coding transcripts in all tissues.

We hypothesise that pathogenic missense variants of *PTCD3* cause a milder form of oxidative phosphorylation deficiency accompanied by symptoms of optic atrophy, ataxia, spasticity, paralysis and polyneuropathy.

## AUTHOR CONTRIBUTIONS

BL and II designed and conceptualised the study. EF, ZK and IM performed clinical analysis of the patient. SP, JS and PZ performed bioinformatics, variant analysis and data analysis. II, NK and HA did molecular biology studies. JAM and MTA did respiratory chain analysis. NTW did in silico modelling. BL, EF, ZK, IM, JS, NTW and II drafted the manuscript. All authors were involved with revising the manuscript.

## FUNDING INFORMATION

This work was supported by European Regional Development Fund [No. 1.1.1.1/18/A/096 ‘The determination of rare inherited diseases' causative mechanisms using whole genome sequencing approach’] and King Salman Center for Disability Research [Research Group No# KSRG‐2022‐105 and KSCDR‐RAC: 2180 004, NK]. SP was supported by the Estonian Research Council grant [PSG774]. This research was funded by U.S. National Science Foundation [MCB‐2024182 to NTW] and the U.S. National Institutes of Health [R15GM148890 to NTW].

## CONFLICT OF INTEREST STATEMENT

The authors declare no conflicts of interest.

## ETHICS STATEMENT

All procedures followed were in accordance with the ethical standards of the responsible committee on human experimentation (institutional and national) and with the Helsinki Declaration of 1975, as revised in 2000 (5). Informed consent was obtained from all patients for being included in the study.

Individuals affected with rare unidentified inherited diseases have been recruited in the Genome Database of the Latvian population (Riga, Latvia) in the framework of the ERDF research project ‘The determination of the causative mechanisms of rare inherited diseases using the whole genome sequencing approach’. The approval of the Latvian Central Committee of Medical Ethics (Protocol No. 2019‐3, Chapter 7, from 30.05.2019), covers all consent and data handling‐related issues for genetic research of the patients involved. Regarding the Saudi patient and his family members, the determination and consenting were carried out under a project (RAC#2120022), which was approved by the institutional review board of the King Faisal Specialist and Research Centre.

## INFORMED CONSENT

Additional informed consent was obtained from all patients for which identifying information is included in this article.

## ANIMAL RIGHTS

This article does not contain any studies with animal subjects performed by the any of the authors.

## Supporting information


**Data S1.** Supporting information.

**Video S1.** Patient, case 1.

**Video S2.** Patient, case 2.

## Data Availability

All data are available upon reasonable request.
