# Supplementary material for: The phenotypic spectrum of PTCD3 deficiency
Source: JIMD Rep. 2024 May 27;65(5):297–304. doi: 10.1002/jmd2.12424 (PMC11558465; doi:10.1002/jmd2.12424)
Supplement: Supplementary file 1 — Data S1. Supporting information. [file JMD2-65-297-s002.docx]

# Supplement 1

# The Phenotypic Spectrum of *PTCD3* Deficiency

Baiba Lace, Eissa Faqeih, Namik Kaya, Zita Krumina, Johannes A. Mayr, Ieva Micule, **Nathan Thompson Wright,** Melanie T. Achleitner, Hanan AlQudairy, Sander Pajusalu, Janis Stavusis, Pawel Zayakin, Inna Inashkina


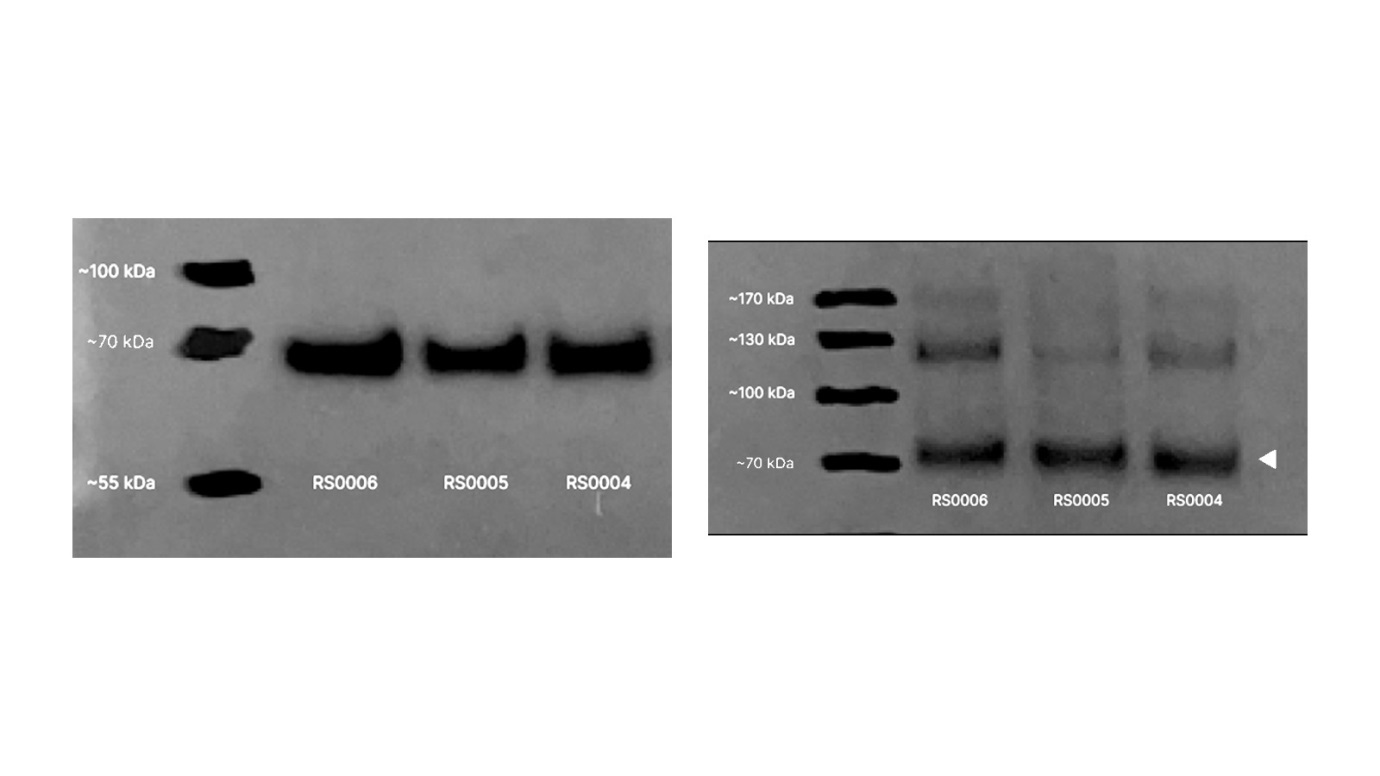


A B

Figure 1. - 8% SDS-PAGE immunoblot calibration of the HSP-60 protein in mitochondrial lysates after normalisation. **B** - 8% SDS-PAGE immunoblot analysis of case 1 (RS0004), case 2 (RS0005) and unaffected mother (RS0006) samples for PTCD3 protein in mitochondrial lysates.

## Methods

*DNA extraction*

Total DNA was extracted from peripheral blood leukocytes using the standard phenol/chloroform method for samples associated with patients 1 and 2. In case of 3, five ml of peripheral blood was used for genomic DNA extraction using the Gentra® Puregene DNA Purification Kit (Gentra Systems, Inc. Minneapolis, MN, USA). DNA concentration was determined using NanoDrop® ND-1000 (NanoDrop Inc., Wilmington, DE, US).

*Massive parallel sequencing*

Cases 1&2. Libraries were prepared from 300 ng of high-quality genomic DNA using the MGIEasy Universal DNA Library Prep Set (MGI Tech Co., Shenzhen, China) following the manufacturer’s instructions. The libraries were sequenced at a mean coverage of 30X on the MGI platform MGISEQ-2000RS, using the nanoball approach and paired-end 150 bp reads.

Case 3. Genomic DNA was enzymatically fragmented. The target regions were enriched using DNA capture probes. These regions include approximately 41 Mb of the human coding exome (target >98% of the coding RefSeq from the human genome build GRCh37/hg19) as well as the mitochondrial genome. The generated library is sequenced on an Illumina platform to obtain at least 20x coverage depth for >98% of the targeted bases.

*Bioinformatics Analyses of Exome Data and Variant Filtering*

Cases 1&2

Fastq files were processed and variants were called using GATK v4 best-practice guidelines using wdl scripts and Cromwell workflow management system ^1^. Shortly, raw sequencing reads were mapped to hg38 reference genome using BWA MEM algorithm (v 0.7.15), followed by the processing using GATK (v4.0.6.0) including flagging duplicated reads and base score. Variants were first called for individual samples using GATK HaplotypeCaller, before multi-sample joint aggregation and re-annotation using GATK GenotypeGVCFs. The variant call set was annotated using hail 0.1 (Hail Team. Hail 0.1. https://github.com/hail-is/hail) and then uploaded to the Seqr platform for collaborative variant analysis. Variant filtration focused on rare variants in coding regions and splice sites predicted to affect protein function and following the known inheritance pattern for the disease-associated genes. Variants were functionally annotated based on data from SiFT ^2^, CADD ^3^, ExAC, 1000genomes and Polyphen 2 ^4^.

*PCR and Sanger Sequencing*

Case 3. Gene specific primers were designed using Primer 3 web tool, the sequences of oligonucleotides are available upon request. Direct sequencing of amplified PCR products was performed on an ABI PRISM 3100 genetic analyser (Applied Biosystems, Foster City, CA, USA).

*Patient-derived Cell Lines*

Cases 1&2

Fibroblasts were isolated from a skin biopsy sample of patients using enzymatic digestion ^5^. The fibroblasts were propagated in DMEM cell culture medium supplemented with 10% foetal bovine serum and 1% penicillin and streptomycin, at 37^0^C, 5% CO_2_ and 95% humidity.

Case 3. A 3 ml peripheral blood sample was collected from the patient in a sodium heparin tube to establish the EBV-transformed lymphoblastoid cell line (LCL). A skin biopsy was collected from the patient to establish a fibroblast cell line (FCL).

*Protein expression analysis*

Case 1&2. The cultivated patients’ and control fibroblasts were harvested and washed with PBS. The cell pellet was suspended in 10 mM Tris (pH 7.4) at a volume ten times that of the exceed cell mass. Subsequently, homogenization was performed on ice using Dounce homogenizers A and B following the manufacturer’ instructions. The cell suspension was then diluted five times with 1.5 M sucrose solution to prevent mitochondrial disruption and centrifuged at 600 g at 4 ° C for 10 minutes. The supernatant, which contained mitochondria, was collected in a fresh tube, and centrifuged at 10,000 g at 4°C for 10 minutes. The resulting pellet was resuspended in 500 µl of SEKT buffer (250 mM sucrose, 2 mM EGTA, 40 mM KCl, 20 mM Tris) and centrifuged at 10,000 g at 4°C for 10 minutes. The pelleted mitochondria were then resuspended again in SEKT buffer, aliquoted, and stored at -80°C for future use. Mitochondria lysates were obtained using Tween reagent (final concentration 1%) on ice, followed by SDS-PAGE and electroblotting. Proteins were visualised by immunostaining with specific antibodies detected fluorometrically using [Amersham ECL detection reagents](https://www.cytivalifesciences.com/redirect/base/P-05842) (Cytiva, Marlborough, MA, USA.). The antibodies used in this study were anti-PTCD3 (HPA041382, Sigma-Aldrich), anti-HSP60 (Abcam, UK).

*RNA extraction and RT-PCR*

Case 3. RNA was extracted from patient lymphoblastoid cell lines using the QIAamp RNA Blood Mini Kit (QIAGEN, Hilden, Germany). To investigate the impact of the splice site variant on the transcript, cDNA was synthesised using the high capacity cDNA reverse transcription kit (Thermo Fisher Scientific Corp., [Waltham, MA](https://en.wikipedia.org/wiki/Waltham,_Massachusetts), U.S.). Gene-specific primers designed to include the variant site and potentially affected regions were used during the amplification of the cDNA. The RT-PCR products were analysed on a 2% agarose gel.

*Determination of the oxygen consumption rate (OCR) by Seahorse XFe96 analyser*

The fibroblasts of the affected individuals and controls were cultured in 75 cm^2^ flasks in DMEM (Sigma-Aldrich; D5648) to 80% confluency. For seeding in a 96-well plate, the media was removed, and the cells were washed once with 5 ml phosphate-buffered saline (PBS). The cells were trypsinized with 5 ml 0.05% trypsin/0.53 mM EDTA for 3 minutes at 37°C and 5% CO_2_ in the incubator. The cell suspension was neutralized in 10 ml DMEM medium in a 15 ml Greiner tube. To remove the media, the suspension was centrifuged at 2,000 rpm for 5 minutes and the supernatant was discarded. The cell pellet was resuspended in 4 ml DMEM, and the cell number was determined with CASY cell counter. For a 96-well plate, 80 µl cell suspension containing 12 000 cells were seeded per well. The plate was incubated for 1 h under lamina flow to allow the cells to attach and build up a uniform cell layer. Overnight, the plate was placed in the incubator at 37 ° C with 5% CO_2_. For calibration, the sensor cartridge was incubated with 200 µl XF Calibrant (Agilent Technologies, pH 7,4) per well at 37°C without CO2 overnight. Seahorse XF Media (Agilent Technologies, pH 7,4) was supplemented with 2 mM L-glutamine, 10 mM glucose and 1 mM sodium pyruvate. Before measurement, DMEM medium of seeded fibroblasts was discarded and cells were washed two times with 120 µl prepared assay medium of 120 l. 180 µl of the seahorse medium was added and the plate was incubated at 37°C without CO2 for 1 h. Oligomycin (50 µM), FCCP (40 µM), antimycin A (5 µM) and rotenone (5 µM) were diluted in assay medium. 20 µl of Oligomycin (Port A), 20 µl of FCCP (Port B) and 20 µl antimycin A together with rotenone (Port C) were injected into the ports of the sensor cartridge. The baseline measurement comprised 4 cycles, which include 3 minutes mixing and 5 minutes measuring for each cycle. After each injection 4 measuring points were recorded, which also comprise 3 minutes mixing and 5 minutes measuring for each cycle. Oxygen consumption rate (OCR) were measured with XF96 extracellular flux analyzer (Seahorse, Agilent) in fibroblasts of two *PTCD3* individuals and controls (OCR in pmol/min/Norm.Unit.). Unit indicates that the measured OCR (pmol/min) is normalized to the cell number (Norm. Unit). Eight replicates for the patient were compared to age matched controls. For statistical analysis of the extracellular flux analysis, a one-way analysis of variance (ANOVA) was used by the program GraphPad Prism 10.

*Computational modelling*

Computational modelling was performed using the human mt-SSU (PDB accession 6GAZ) ^6^.  The PTCD3 subunit was removed from the larger structure and equilibrated for 85 ns in explicit solvent at 37 °C, 150 mM NaCl using the Amber14 force field in the program YASARA ^7^. The His269Tyr mutation was constructed using the ‘swap’ command and equilibrated for 73 ns. Simulations were surveyed every 100 ps and analysed using standard YASARA macros as previously described ^8^.

Video <https://drive.google.com/drive/u/0/folders/1K3CZ7pAB9TJMRnyKBVRSGEIjWmxazU2->

References

1. Institute, B. Cromwell workflow management system. https://cromwell.readthedocs.io.

2. Kumar, P., Henikoff, S. & Ng, P. C. Predicting the effects of coding non-synonymous variants on protein function using the SIFT algorithm. *Nat. Protoc.* **4**, 1073–1081 (2009).

3. Kircher, M. *et al.* A general framework for estimating the relative pathogenicity of human genetic variants. *Nat. Genet.* **46**, 310–315 (2014).

4. Adzhubei, I., Jordan, D. M. & Sunyaev, S. R. Predicting Functional Effect of Human Missense Mutations Using PolyPhen‐2. *Curr. Protoc. Hum. Genet.* **76**, (2013).

5. Mak, S. K., Tewari, D., Tetrud, J. W., Langston, J. W. & Schüle, B. Mitochondrial Dysfunction in Skin Fibroblasts from a Parkinson’s Disease Patient with an alpha-Synuclein Triplication. *J. Parkinsons. Dis.* **1**, 175–183 (2011).

6. Kummer, E. *et al.* Unique features of mammalian mitochondrial translation initiation revealed by cryo-EM. *Nature* **560**, 263–267 (2018).

7. Krieger, E. & Vriend, G. YASARA View—molecular graphics for all devices—from smartphones to workstations. *Bioinformatics* **30**, 2981–2982 (2014).

8. Ng, R. *et al.* Patient mutations linked to arrhythmogenic cardiomyopathy enhance calpain-mediated desmoplakin degradation. *JCI Insight* **4**, (2019).
